# Colistin Resistance *mcr-1*-Gene-Bearing *Escherichia coli* Strain from the United States

**DOI:** 10.1128/genomeA.00898-16

**Published:** 2016-09-01

**Authors:** Richard J. Meinersmann, Scott R. Ladely, Jodie R. Plumblee, M. Carolina Hall, Sheron A. Simpson, Linda L. Ballard, Brian E. Scheffler, Linda L. Genzlinger, Kimberly L. Cook

**Affiliations:** a USDA Agricultural Research Service, Athens, Georgia, USA; b USDA Food Safety and Inspection Service, Athens, Georgia, USA; c USDA Agricultural Research Service, Stoneville, Mississippi, USA

## Abstract

Transmissible colistin resistance in the form of an *mcr-1*-gene-bearing plasmid has been recently reported in *Enterobacteriaceae* in several parts of the world. We report here the completed genome sequence of an *Escherichia coli* strain isolated from swine in the United States that carried the *mcr-1* gene on an IncI2-type plasmid.

## GENOME ANNOUNCEMENT

Colistin is a reserved antimicrobial agent that is used to treat multidrug-resistant infections. Resistance to colistin is known to be innate among Gram-positive organisms and conferred by chromosomal modifications that alter the drug target in Gram-negative species. Liu et al. (1) recently described a gene, *mcr*-1, which codes for a phosphoethanolamine transferase enzyme, which catalyzes a change in the colistin target conferring resistance and was carried on an IncI2 plasmid with high conjugation efficiency. Since then, the gene has been detected in isolates recovered from food animals around the world (2–4) and from human patients (2, 5, 6).

In collaboration with National Antimicrobial Resistance Monitoring System (NARMS), we began a search for the resistance gene in food animals within the United States. Aliquots of NARMS cecal samples (from chicken, turkey, cattle, and swine) were incubated overnight at 37°C in buffered peptone water (Acumedia; Neogen Corporation) with 2 µg/ml colistin (Sigma-Aldrich), followed by screening for the *mcr-1* gene using PCR, as described by Liu et al. (1).

Cultures producing a PCR amplification product of the expected size were plated onto MacConkey agar (Acumedia) supplemented with 2 µg/ml colistin for isolation. Presumptive positive colonies were rescreened by PCR and were identified to the species level using the Vitek 2 system (bioMérieux).

A colistin-resistant isolate identified as *Escherichia coli* from a pig from South Carolina was found to be PCR positive for the *mcr-1* gene. DNA was prepared from the isolate, and the genomic sequence of the strain was determined by sequencing with Pacific Biosciences XL-C2 chemistry and assembled with CANU assembler version 1.3 (7) into a circular chromosomal contig of 5,005,730 bases and five circular plasmid contigs of 65,889 (pSLy1), 129,035 (pSLy2), 114,472 (pSLy3), 100,096 (pSLy4), and 9,580 (pSLy5) bases. These sequences were used as a scaffold for assembling data from three runs on an Illumina MiSeq, which was used to edit the contig. The mean Illumina data coverage for the chromosome was 62-fold and exceeded 170-fold for all the plasmids.

The chromosome carried genes for multilocus sequence type (MLST) ST3234 (8), genes for serotype O160:H40, and the virulence factors *astA* and *lpfA* (http://www.genomicepidemiology.org). Antimicrobial resistance genes *strA*, *strB*, *sul2*, and *tet*(A) were also found on the chromosome. pSLy1 carried an IncI2 replication initiation protein gene and carried the *mcr-1* gene that was 100% identical to all the *mcr-1* genes found in GenBank. pSLy2 was an IncFIB plasmid that carried *oqxA*, *oqxB*, and *bla*_TEM_. pSLy4 was an IncI1 plasmid with no known antimicrobial resistance gene. No known replication initiation protein gene or antimicrobial resistance genes were detected on pSLy3 and pSLy5.

This isolate is different from any other strain that has been described as carrying the *mcr-1* gene. The finding of 100% identity of all *mcr-1* genes gives no phylogenetic signal. Therefore, tracing the lineage of the *mcr-1*-gene-carrying plasmids will require more sequences of IncI2 plasmids.

### Accession number(s).

Sequences were deposited in GenBank under the following accession numbers: CP015912 (chromosome), CP015913 (pSLy1), CP015914 (pSLy2), CP015915 (pSLy3), CP015916 (pSLy4), and CP015917 (pSLy5).
